# Analysis of the Antifungal Properties of *Bacillus velezensis* B-4 Through a Bioassay and Complete-Genome Sequencing

**DOI:** 10.3389/fgene.2020.00703

**Published:** 2020-07-16

**Authors:** Zheyuan Zhu, Qiong Peng, Yilong Man, Zuren Li, Xiaomao Zhou, Lianyang Bai, Di Peng

**Affiliations:** ^1^College of Plant Protection, Hunan Agricultural University, Changsha, China; ^2^Hunan Agricultural Biotechnology Research Institute, Changsha, China; ^3^Hunan Academy of Agricultural Sciences (CAAS), Changsha, China

**Keywords:** antibacterial activity, complete genome, comparative genome, *Bacillus velezensis* B-4, biology control

## Abstract

The strain B-4, isolated from a field in Changsha (China), presents strong antifungal activities, as identified by the Kirby–Bauer test, especially for pathogens that harm crops. Here, we obtained the complete genome sequence of the strain B-4 by Pacific Biosciences single-molecule real-time sequencing, making it well analyzed for understanding mechanisms and creating biological agents. Its 3,919-kb circular chromosome genome has 3,725 protein-coding genes [coding sequences (CDSs)] and 46.7% guanine–cytosine content. A comparative genome analysis of B-4 with other published strains (including *Bacillus velezensis*, *Bacillus amyloliquefaciens*, and *Bacillus subtilis*) revealed that the strain B-4 is a *B. velezensis* strain. These different strains have 2,889 CDSs in common, whereas 179 CDSs were found to be unique in the strain B-4, which is a far greater number than that in other strains. Regarding the antifungal activities of B-4, we were specifically concerned with the genes involved in the biosynthesis of secondary metabolites. In total, more than 19.56% of the genome was annotated to 12 gene clusters relating to synthesis of antimicrobial metabolites, which contained various enzyme-encoding operons for non-ribosomal peptide synthetases, polyketide synthases, and lantipeptide synthesis proteins. They were all considered to be related to the production of bacteriostatic substances or stimulation of induced systemic resistance by bacterial metabolites. These situations also present an advantage over those of other strains for biocontrol potential. We provide evidence that the biological control effect of the strain B-4, as demonstrated in antibacterial activity experiments and predicted from the complete genome sequence analysis, provides the basis for research promoting agricultural research on sustainable development, especially the contribution of biotechnology to agriculture.

## Introduction

Control of crop diseases has been an important process in agriculture production. Chemical pesticides have been used to control crop disease in popular and efficient ways, but to achieve highly effective control efficiency, pesticides have always been overdosed by practitioners who work in agriculture, which has had a detrimental effect on the environment or has caused crops to become resistant (Chang, 2018). With increasing requirements for the quality of food safety, the protection of crops cannot depend on chemical pesticides (Syed Ab Rahman et al., 2018). For many years, antagonistic bacteria have been used to control crop diseases caused by fungi, bacteria, viruses, or nematodes. This method can be classified as biological control and has received unusual attention because of its safety and environmentally friendly impact on crops (Fernando et al., 2007; Beneduzi et al., 2012).

*Bacillus velezensis* is a species that differs from *Bacillus amyloliquefaciens* but was previously reported as a later heterotypic synonym. The use of *B. velezensis* in biological control research has become popular. Moreover, *Bacillus* sp. are considered to have extensive bacteriostatic properties; this species has been reported to produce a variety of lipoprotein antibacterial substances, such as fengycin (Ongena et al., 2007). Using confocal scanning laser microscopy to analyze *Arabidopsis* root surfaces treated with *Bacillus subtilis* 6051, a stable, extensive biofilm and secreted surfactin were noted to combat exogenous pathogens (Bais et al., 2004). The reason that *Bacillus* sp. can produce antibiotics via a secondary metabolic activity is related to genes and proteins. *B. velezensis* FZB42 is an environmental bacterium that suppresses competitive harmful organisms. These activities were assigned to complete polyketide synthase (PKS) gene clusters, and it was founded that the pks1 (*bae*) and pks3 (*dif*) gene clusters encode proteins necessary for the biosynthesis of the polyene antibiotics (Chen et al., 2006). Molecular biological techniques should be useful for understanding the mechanism of bacterial resistance to diseases at the gene level. A recent example, *B. velezensis* M75, was isolated from cotton waste; the authors used the complete genome sequence and found that the genome contained operons that are related to the biosynthesis of secondary metabolites (Kim et al., 2017). In conclusion, ample gene coding is related to the mechanism of an antimicrobial activity, and complete-genome sequencing technology could help to better study biological control. Although many research results have suggested that bacteria play a major role in biological control, which could inhibit the growth of other strains, the results concerning the mechanism of action and interpretation at the genetic level remain inconclusive, and the complete genomes of only a limited number of antagonistic bacteria have been determined.

In this study, *B. velezensis* B-4 was isolated from a field in Changsha, China. We identified and reported a *B. velezensis* B-4 antibacterial activity and complete genome sequencing results. Interestingly, the strain B-4 could be developed as a novel biocontrol agent on the basis of the actual bacteriostatic tests and gene features.

## Materials and Methods

### Strains, Fungi, and Culture Media

Here, the strain B-4 was newly isolated from a field, and as a bacterial strain, it was obtained with GenBank ID (CP031424.1). *Sclerotinia sclerotiorum*, *Thanatephorus cucumeris*, and *Fusarium graminearum* were kept in the microbiology laboratory of *Hunan Agricultural Biotechnology Research Institute* (Changsha, China). The Luria–Bertani (LB) medium comprised 2.5 g of yeast extract, 5 g of peptone, 5 g of NaCl, and 500 ml of distilled water (pH 7.0). The center of potato dextrose agar (PDA) medium comprised potato 200 g, glucose 20 g, and agar 15 g dissolved in 1,000 ml of distilled water. All of these media were maintained for 30 min in 121°C.

### Bacterial Growth Curve and the Linear Regression Analysis

The B-4 strain [2 ml, bacterial suspension recovered from 4°C, optical density (OD) 600 = 0.322] was grown in LB medium (98 ml) on a shaker incubator (200 rpm) at 28°C, using the UV spectrophotometer measured OD (λ = 600 nm) every hour. At the same time, the number of plate colonies was determined by colony counting method. There were constructed by GraphPad Prism (version 8.3.1) that concluded the strain B-4 bacterial growth curves and the linear regression between OD and number of living bacterium (Zuluaga et al., 2009).

### Evaluation of the Antifungal Activities of the Strain B-4

We evaluated whether the strain B-4 has a strong antimicrobial effect for several plant pathogenic bacteria by the Kirby–Bauer (K-B) method (Gopinath et al., 2012; Gautam et al., 2013).

The K-B method was utilized to assess the activity of the strain B-4 against plant pathogenic bacteria, including but not limited to *S. sclerotiorum*, *T. cucumeris*, and *F. graminearum*. The B-4 strain was grown in LB medium on a shaker incubator (200 rpm) at 28°C. After 40 h, fungus was inoculated on the center of PDA plates. An 8-mm susceptibility paper disk was placed on the PDA plates for the inoculation of the strain B-4. Ten microliters of fermentation broth (72.61 × 10^9^ CFU/ml) of the strain B-4 was then inoculated into the paper disk. After the completion of the above process, the plates were incubated at 25°C for 2–3 days, and the zone of inhibition was observed. Meanwhile, the control group, with plates that did not receive a drop of the fermentation broth of the strain B-4 and only contained the pathogenic bacteria, was incubated in the same environment as the test group. Finally, the Fisher least significant difference method was used to analyze the significance of the difference through R software (version 3.6.3).

### Complete-Genome Sequencing and Assembly

The complete genome of the strain B-4 was sequenced by Pacific Biosciences (PacBio) single-molecule real-time (SMRT) sequencing technology with an approximately 15-kb SMRTbell^TM^ template library (MajorBio Co., Shanghai, China). Sequencing raw data were analyzed after quality inspection (Watson, 2014). To create a high-quality genomic sequence, all reads were spliced into contigs and then combined into scaffolds that did not include errors and gaps.

The genome sequences were submitted to a bioinformatic analysis to further reveal biological information. Therefore, in this study, Barrnap 0.4.2 and tRNAscan-SE v1.3.1 were used to predict the rRNA and tRNA genes, respectively (Lowe and Eddy, 1997). The prediction of the strain B-4 genes was carried out using Glimmer 3.02.

### Complete-Genome Annotation and Comparative Genomic Analysis

All of the genes from the strain B-4 were annotated by BLAST 2.2.28 + program^1^, covering the Nr, Genes, STRING, and Gene Ontology (GO) databases (Ashburner et al., 2000; Kanehisa et al., 2016; O’Leary et al., 2016; Szklarczyk et al., 2017). In a comparative genomic analysis, firstly, all of genes were categorized into different types by the Clusters of Orthologous Groups of proteins (COGs) database, and then a private project was constructed comprising *B. velezensis* FZB42 (NC_009725.1), *B. velezensis* M75 (NZ_CP016395.1), *B. amyloliquefaciens* DSM7 (NC_014551.1), *B. amyloliquefaciens* Y2 (NC_017912.1), *B. amyloliquefaciens* CC178 (NC_022653.1), *B. amyloliquefaciens* MT45 (NZ_CP011252.1), *B. subtilis* 168 (NC_000964.3), and the strain B-4, with known genome sequences. To construct a phylogenetic tree by RAxML for this project, OrthoMC software was used to determine the core and specific genes of the genomes (Li et al., 2003; Stamatakis, 2006). In the following step, a comparative circular genome map was created using BRIG (BLAST Ring Image Generator, 0.95-dev.0004, Alikhan et al., 2011), and Mauve (version 2.4.0, Darling et al., 2004) was used to evaluate synteny. We obtained gene clusters for secondary metabolite biosynthesis using the antiSMASH3.0 program and using Blast with other strains (Weber et al., 2015).

## Results

### Bacterial Growth Pattern of the Strain B-4

**FIGURE 1:**
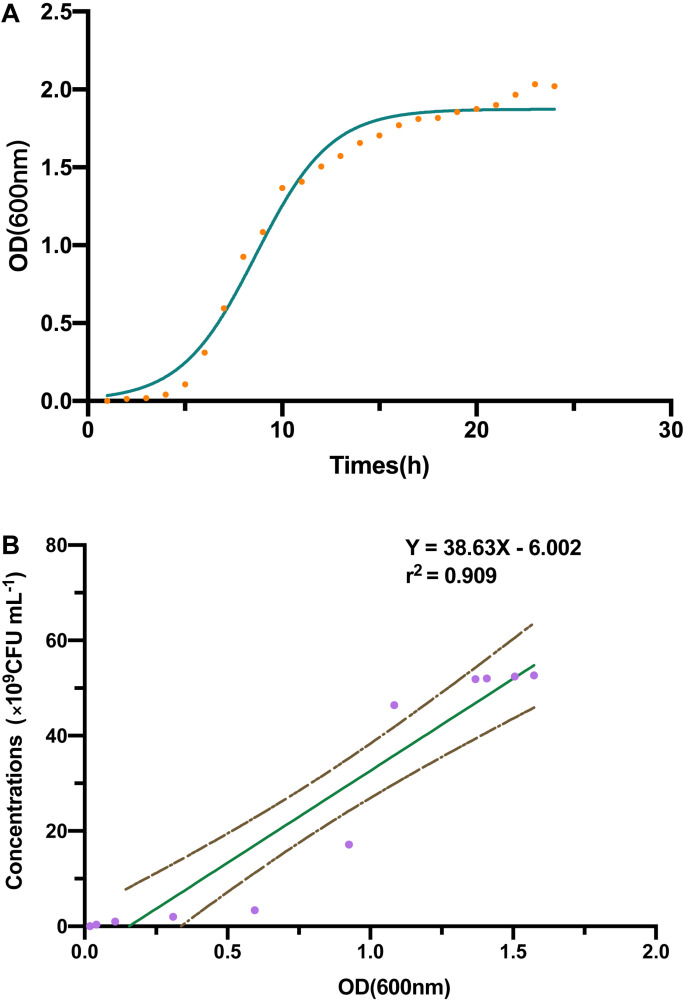
**(A)** The strain B-4 growth curve. **(B)** The linear regression between optical density (OD) and number of living bacterium.

Through a curve fitting analysis, we constructed bacterial growth curve (Figure 1A), which confirmed the exponential phase of the strain B-4 from 4 to 15 h. During this phase, we analyzed the linear regression equation and correlation coefficient between the OD of fermentation broth and the number of living bacterium (Figure 1B).

### Antifungal Activities of the Strain B-4

**FIGURE 2:**
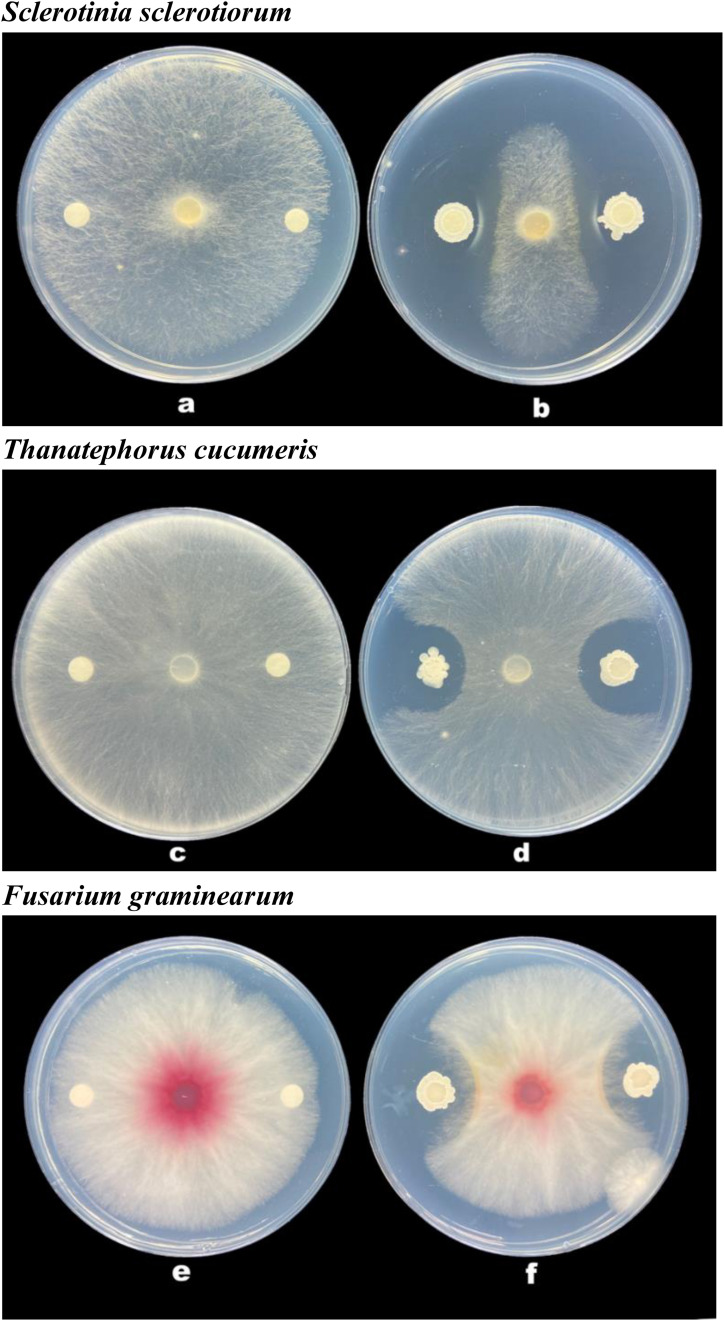
Antifungal activity of *Bacillus velezensis* B-4 against fungus. **(a,c,e)** Disk paper with Luria–Bertani (LB) medium added. **(b,d,f)** Disk paper with the strain B-4 fermentation broth.

The strain B-4 showed excellent antifungal activities against pathogenic fungus. We measured the linear distance from the center of the susceptibility paper to the edge of the pathogen as the bacteriostatic radius and measured colony growth radius of the fungus. The mean inhibitory zone of the four replicates was measured, and the average amount was calculated. From Table 1, compared with the control, the strain B-4 had a significant antifungal activity. In the experimental group, the strain B-4 formed a very clear blank growth area between the disk with the bacterial suspension and the pathogenic bacteria (Figure 2). As expected, the strain B-4, which acts mainly against pathogenic bacteria, had a growth advantage in a flat confrontation test.

**TABLE 1:** The effect of the strain B-4 on fungus growth.

	**Colony radius (mm)**		**Inhibitory zone radius (mm)**	
	**LB 10 μl**	**B-4 10 μl**	**LB 10 μl**	**B-4 10 μl**
*Sclerotinia sclerotiorum*	31.8 ± 0.4a	13.8 ± 1.0b	0.0b	11.7 ± 0.8a
*Thanatephorus cucumeris*	45.0a	15.8 ± 2.1b	0.0b	9.8 ± 0.5a
*Fusarium graminearum*	35.3 ± 2.1a	16.8 ± 1.8b	0.0b	10.75 ± 0.4a

*Different letters in the same rows indicate significant difference at the 0.05 level. And results are means ± standard error (SE) of values.*

### Genome Sequencing and Phylogenomic Analysis of the Strain B-4

**FIGURE 3:**
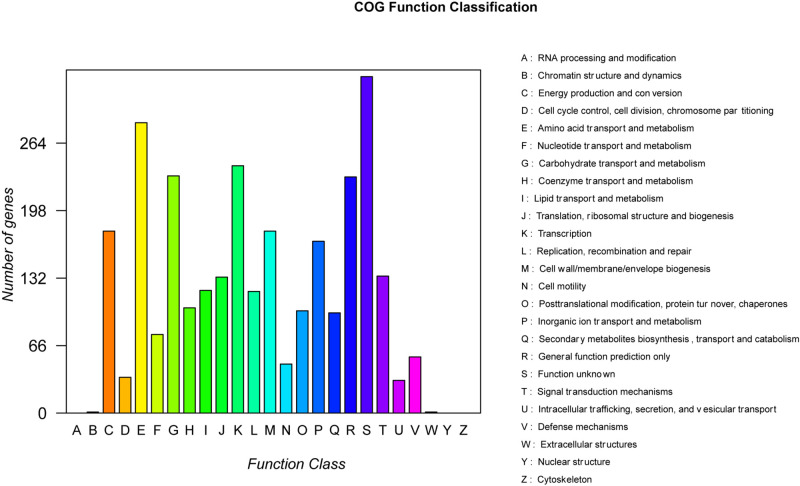
The statistical results of the Clusters of Orthologous Groups of proteins (COG) functional classification of genomic protein.

A total of 99,637 reads were sequenced, and the average length of reads was 5,904 bp. These reads were assembled to a circular chromosome of the strain B-4 genome with a size of 3,919,798 bp and a guanine–cytosine (GC) content of 56.67% (Table 2). The chromosome contains 4,127 predicted genes [4,014 coding sequences (CDSs), 27 rRNAs, and 86 tRNAs]. In addition, different functions of genes were classified based on a COG analysis, which revealed a large proportion of “metabolism” (43.48%); this category is considered to be related to predicted proteins for a secondary metabolism activity. Among them, 1,260 proteins were classified into eight COG functions (Figure 3), and in particular, 7.78% of proteins were related to “secondary metabolites biosynthesis, transport and catabolism.” This situation was more considerable than that in other strains and was strong evidence of the antifungal properties of the strain B-4.

**TABLE 2:** Genomic features of *Bacillus velezensis* B-4.

**Features**	**Value**
Gene total length (bp)	3,919,798
Gene average length (bp)	870
Chromosome number	1
GC content in gene region (%)	46.7
Protein-coding genes (CDSs)	3,725
rRNA genes	27
tRNA genes	86

**FIGURE 4:**
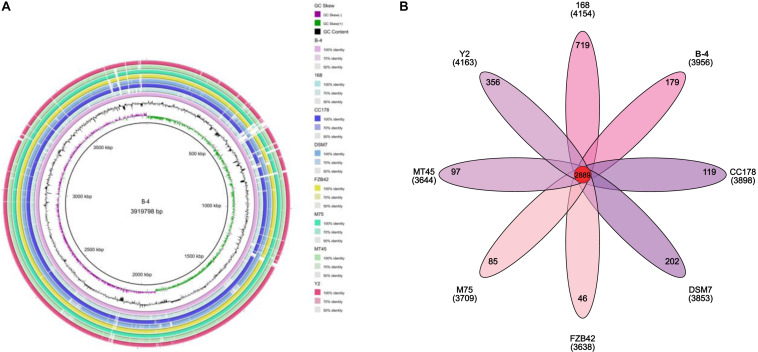
Comparative genome analysis. **(A)** Comparative circular genome map. The figure compares multiple genomes, locates genes in other genomes through known genome information according to the homology of coding order and structure, and summarizes the potential functions of each gene and the changes in the internal structure of the genome. **(B)** Comparative genome analysis based on protein cluster analysis shows shared and unique protein clusters.

**FIGURE 5:**
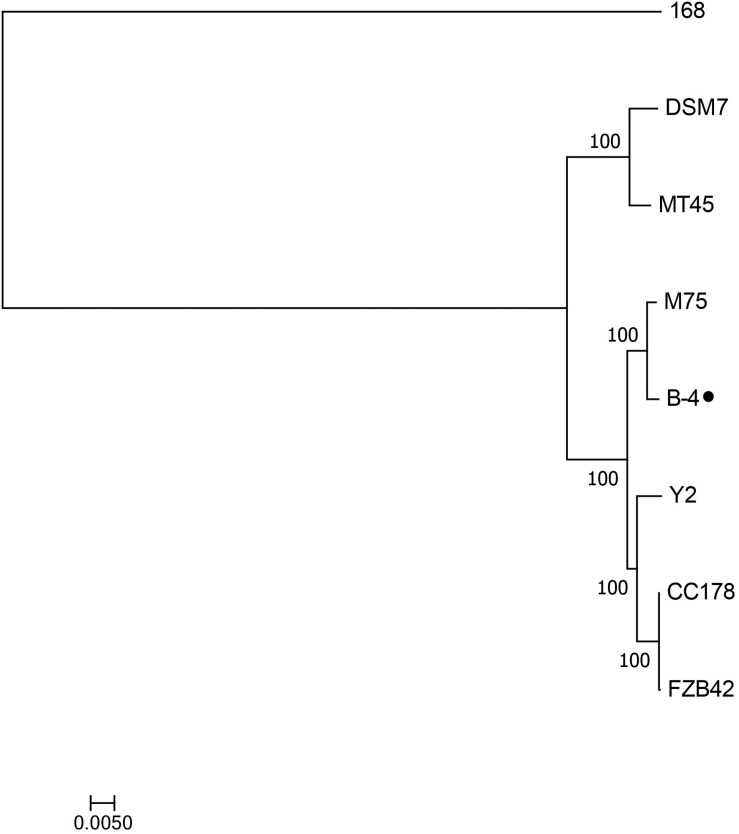
Phylogenetic tree based on whole genome showed the phylogenetic position of the strain B-4 (⚫) and that the strain B-4 belonged to *Bacillus velezensis*. And the *Bacillus subtilis* 168 (NC_000964.3) was used as the out group.

The genome of the strain B-4 was compared with that of other strains of the same genus (*Bacillus* sp.). Based on the results from OrthoMC software, the gene numbers of B-4 were found to be higher than those of the strains M75, DSM7, CC178, and MT45; and the number of core genes was 2,889, with all compared strains containing the strains M75, DSM7, CC178, MT45, Y2, and 168. Additionally, 179 CDSs of the strain B-4 were singletons. Among these genes, 33.5% were annotated to hypothetical proteins with unknown functions (Figure 4). Then, to classify the strain B-4, a phylogenomic tree of the complete genome was constructed and compared with closely related genomes. Based on the analysis results, the strain B-4 formed a close genetic relationship with the strain M75 and showed a distinct branch from the strains 168, DSM7, CC178, MT45, and Y2 (Figure 5). Therefore, the strain B-4 is a newly found antifungal strain recognized as *B. velezensis*.

In an analysis of the gene clusters, 12 secondary metabolite gene clusters of *B. velezensis* B-4 were identified by the antiSMASH (version 4.0.2) database (Table 3). Those included one Nrps, one terpene, two transatpks, two transatpks-Nrps, one terpene, one T3pks, one bacteriocin-Nrps, and one lantipeptide clusters, and most of them were related to the production of non-ribosomal peptide synthetase (NRPS) and PKS systems. In comparison with other strains published, clusters 2 and 12 were unique metabolite pathways related to butirosin and mersacidin, respectively.

**TABLE 3:** Gene clusters involved in synthesis of secondary metabolites in *Bacillus velezensis* B-4.

**Cluster**	**Type**	**Size (bp)**	**Compound**	**Similarity (%)**	**MIBiG BGC-ID**
1	Nrps	65,407	Surfactin	82	BGC0000433_c1
2	Otherks	41,244	Butirosin	7	BGC0000693_c1
3	Terpene	20,740	–	–	–
4	Transatpks	85,884	Macrolactin	100	BGC0000181_c1
5	Transatpks-Nrps	102,704	Bacillaene	100	BGC0001089_c1
6	Transatpks-Nrps	137,834	Fengycin	100	BGC0001095_c1
7	Terpene	21,883	–	–	–
8	T3pks	41,100	–	–	–
9	Transatpks	100,432	Difficidin	100	BGC0000176_c1
10	Bacteriocin-Nrps	66,792	Bacillibactin	100	BGC0000309_c1
11	Other	41,418	Bacilysin	100	BGC0001184_c1
12	Lantipeptide	23,188	Mersacidin	90	BGC0000527_c1

## Discussion and Conclusion

Research on the strain of *B. velezensis* B-4 showed outstanding growth rate inhibition of *S. sclerotiorum*, which is a devastating necrotrophic plant pathogen. Simultaneously, farmers use high levels of carbendazim to control classic crop disease pathogens including *S. sclerotiorum*, *T. cucumeris*, and *F. graminearum*; and high resistance has been reported in China (Wang et al., 2014). High-dose applications are accompanied by excessive pesticide residues in the environment, impacting the health of humans and animals. Rape, rice, and wheat are very important crops but are affected by diseases all year round; fortunately, the effective inhibition radius of *B. velezensis* B-4 on *S. sclerotiorum*, *T. cucumeris*, and *F. graminearum* reached 11.7 ± 0.8, 9.8 ± 0.5, and 10.75 ± 0.4 mm separately, which was the same as that of *Bacillus* sp., which have the ability to inhibit the growth and to proliferate harmful bacteria, pathogenic bacteria, and so forth. Through bioassay experiments developed in our laboratory, *B. velezensis* B-4 was confirmed to be an ideal biological control agent to meet the needs of sustainable farmland development.

The complete-genome sequencing and comparative genomics analysis provided further molecular biology evidence for inhibiting pathogens with *B. velezensis* B-4. Microbial genome sequencing produces numerous sequences of deduced proteins, and information about these proteins can also be annotated by the COGs database, which includes a detailed prediction of the biological function(s) of representative complete genomes from all bacteria (Galperin et al., 2015). Specifically, the complete genome sequence of *B. velezensis* B-4 was grouped by category using the COG database. In all, 43.48% of its genome is devoted to the process of metabolism, including 7.78% participating in secondary metabolite biosynthesis, transport, and catabolism. These results further support the ideal biocontrol function of *B. velezensis* B-4. There are also other possible analyses. A GO database annotation makes it easier for us to understand the biological meaning behind genes (Götz et al., 2008; Pusztahelyi et al., 2015). The genome annotation of the metabolic process category had an absolute enrichment in the GO database, which may be an important factor in the inhibition of pathogens. In addition, some types of genomes with a number of beneficial traits, such as cellular process, single-organism process, and binding and catalytic activities, showed that the activity of *B. velezensis* B-4 was concentrated mainly in differentiation, enzyme catalysis, and metabolism. However, further investigations combining two categories of annotation methods found that a portion of the genome was not annotated. Relative to other strains, *B. velezensis* B-4 contains a large number of unique genes that were not found in the compared strains, which showed that *B. velezensis* B-4 was unique and had development potential. We suggest that these genes also have general effects on the physiological activity of the strain. For instance, 11.35% of the genome was not annotated in the COG database, which could be involved in the activity of inhibiting pathogenic bacteria. Taken together, these findings contribute to our understanding of *B. velezensis* B-4, which has antibacterial effects, on the basis of comparative genomics, and it contains numerous unknown genome regions that can be developed.

The comparative genomics analysis of the complete genome of *B. velezensis* B-4 revealed that it had excellent antibacterial effects. Moreover, recent research has shown that activities of biocontrol bacteria are closely related to secondary metabolites that have no clear function in life activities (Guo et al., 2013; Chowdhury et al., 2015). According to gene cluster research in *Bacillus* sp., various antifungal materials are produced by *Bacillus* sp. For example, a study of *B. subtilis* 916 demonstrated that bacillomycin Ls and fengycins produced by the strain contributed mainly to an antifungal activity (Luo et al., 2015). In addition, *B. velezensis* LS69 was found to have a series of gene clusters relevant to the activity against pathogenic bacteria (Liu et al., 2017, p. 916). Thus, the search for secondary metabolites in the complete genome of *B. velezensis* B-4 confirmed that it possessed an antagonistic activity. Here, we found four gene clusters playing a major role in non-ribosomal synthesis and three gene clusters involved in PKS, as well as a biosynthetic gene cluster for mersacidin, which is a type of lantipeptide belonging to ribosomally synthesized natural products (RNPs). In fact, secondary metabolites such as fengycins and surfactin are also the major compounds involved in the inhibitory activity. The above substances belong to lipopeptides, which constitute one of the main classes of biosurfactants. González-Jaramillo et al. (2017) evaluated the *B. subtilis* EA-CB0015 strain and found that iturin A and fengycin C inhibited mycelial growth and ascospore germination of *Mycosphaerella fijiensis*, whereas surfactin was not effective. However, we detected a great quantity of gene clusters, showing that *B. velezensis* B-4 had an antibacterial activity; among them, seven gene clusters were corroborated to separately produce macrolactin, bacillaene, fengycin, difficidin, bacillibactin, and bacilysin, which were 80–100% similar to those of others that are known. Four metabolites (bacilysin, bacillibactin, fengycin, and surfactin) produced via NRPS and three metabolites (bacillaene, difficidin, and macrolactin) produced via PKS were reported to be responsible for well-known antimicrobial activities. In contrast to other bacteria, such as the strain DSM7, strain FZB42, or strain M75, there was no lantipeptide-type gene cluster, which is often well known for potent antibacterial activities (Kim et al., 2017). According to alignment, the biosynthetic enzymes for the secondary metabolites bacilysin (BGC0001184_c1), bacillaene (BGC0001089_c1), bacillibactin (BGC0000309_c1), difficidin (BGC0000176_c1), fengycin (BGC0001095_c1), macrolactin (BGC0000181_c1), and mersacidin (BGC0000527_c1) were highly homologous among all the strains. The surfactin-containing gene cluster (involves four genes: *srf*AA, AB, AC, and ATE) had a high level of similarity to that in the others, but other components of genes showed significant differences compared with those of other strains. Interestingly, mersacidin biosynthetic gene clusters (BGC0000527_c1), which had 90% of the genes, showed similarity only in the strain B-4. In addition, mersacidin, as an aminoglycoside antibiotic metabolite, was found in the genome of the strain B-4, which showed a similarity of 7% (BGC0000693_c1). However, there were still some genetic clusters that had been detected or annotated with a function. Similarly, according to Nodwell, it is of tremendous clinical use to analyze bacterial specialized metabolites as bioactive molecules, combined with transcript elongation and other methods (Nodwell, 2017). Therefore, the secretion of secondary metabolites produced by *B. velezensis* B-4 against pathogenic bacteria was confirmed at the level of gene clusters and from additional information that was revealed.

Based on all the research on *B. velezensis* B-4, including evaluation of antifungal activities, complete-genome sequencing by Pacific Biosciences SMRT, and data analysis by comparative genomics, *B. velezensis* B-4 was shown to belong to the antagonist bacteria and to have an excellent antifungal activity against pathogens. Therefore, *B. velezensis* B-4 can be considered a promising biocontrol agent for biological control in crop production. Furthermore, the unknown genes that we found via complete-genome sequencing need to be studied. The results provide useful genomic information that may affect the development of biocontrol agents and the elucidation of functions of *Bacillus* sp. genome.

## Data Availability Statement

The datasets generated for this study can be found in the *B. velezensis* (CP031424.1), *B. velezensis* FZB42(NC_009725.1), *B. velezensis* M75(NZ_CP016395.1), *B. amyloliquefaciens* DSM7(NC_014551.1), *B. amyloliquefaciens* Y2(NC_017912.1), *B. amyloliquefaciens* CC178(NC_022653.1), *B. amyloliquefaciens* MT45(NZ_CP011252.1), and *B. subtilis* 168(NC_000964.3).

## Author Contributions

ZZ selected *Bacillus velezensis* B-4, wrote the manuscript, carried out experimental work, and analyzed the complete genomics sequencing data. QP and YM helped in the bioassay experiments. LB and XZ supervised the entire study. DP helped with the design of the experiments and revised the manuscript. All authors contributed to the article and approved the submitted version.

## Conflict of Interest

The authors declare that the research was conducted in the absence of any commercial or financial relationships that could be construed as a potential conflict of interest.

## Footnotes

1 http://blast.ncbi.nlm.nih.gov/Blast.cgi
